# Supplementary material for: What does it take to learn the rules of RNA base pairing? A lot less than you may think
Source: bioRxiv. 2025 Aug 2:2025.07.31.668042. Preprint. [Version 1] doi: 10.1101/2025.07.31.668042 (PMC12324431; doi:10.1101/2025.07.31.668042)
Supplement: Supplement 1 [file media-1.gz › supplemental_material/src/rscape_v2.6.0.tar.gz!/rscape_v2.6.0/lib/infernal/Manuscripts/banded-cyk/tbl-rmark.doc]

| Rfam 7.0 | family | # | # | avg len |  | non-banded | QDB () | | | QDB () | | | |
| --- | --- | --- | --- | --- | --- | --- | --- | --- | --- | --- | --- | --- | --- |
| ID | name | query | test | query | W | time | time | | spd up | MER | FP | FN | thr |
| RF00177 | SSU_rRNA_5 | 145 | 21 | 593 | 690 | 96.97 | 7.61 | | 12.74 | 0 | 0 | 0 | 9.90 |
| RF00024 | Telomerase-vert | 20 | 11 | 436 | 505 | 52.99 | 4.44 | | 11.94 | 0 | 0 | 0 | 11.30 |
| RF00011 | RNaseP_bact_b | 30 | 1 | 366 | 441 | 33.44 | 3.72 | | 8.98 | 0 | 0 | 0 | 11.31 |
| RF00018 | CsrB | 8 | 1 | 351 | 403 | 28.76 | 2.27 | | 12.68 | 0 | 0 | 0 | 12.98 |
| RF00040 | rne5 | 6 | 1 | 338 | 368 | 19.69 | 2.60 | | 7.57 | 0 | 0 | 0 | 11.79 |
| RF00023 | tmRNA | 19 | 40 | 334 | 463 | 24.58 | 2.47 | | 9.95 | 11 | 0 | 11 | 11.20 |
| RF00010 | RNaseP_bact_a | 233 | 1 | 332 | 514 | 33.82 | 3.29 | | 10.27 | 0 | 0 | 0 | 12.61 |
| RF00009 | RNaseP_nuc | 26 | 21 | 320 | 530 | 27.89 | 7.11 | | 3.93 | 19 | 0 | 19 | 11.67 |
| RF00017 | SRP_euk_arch | 28 | 21 | 303 | 328 | 14.78 | 2.70 | | 5.48 | 6 | 0 | 6 | 10.40 |
| RF00028 | Intron_gpI | 5 | 24 | 300 | 381 | 17.55 | 3.59 | | 4.88 | 19 | 2 | 17 | 10.70 |
| RF00373 | RNaseP_arch | 20 | 13 | 290 | 337 | 15.06 | 3.12 | | 4.82 | 0 | 0 | 0 | 12.23 |
| RF00030 | RNase_MRP | 18 | 3 | 284 | 394 | 19.75 | 3.12 | | 6.34 | 3 | 0 | 3 | 12.46 |
| RF00101 | SraC_RyeA | 6 | 1 | 250 | 278 | 9.50 | 1.54 | | 6.19 | 0 | 0 | 0 | 11.88 |
| RF00230 | T-box | 10 | 35 | 244 | 298 | 8.23 | 1.80 | | 4.58 | 1 | 0 | 1 | 12.34 |
| RF00448 | IRES_EBNA | 7 | 1 | 213 | 238 | 7.25 | 1.26 | | 5.76 | 1 | 0 | 1 | 11.99 |
| RF00012 | U3 | 6 | 5 | 212 | 240 | 7.17 | 1.61 | | 4.46 | 2 | 0 | 2 | 13.02 |
| RF00174 | Cobalamin | 87 | 66 | 203 | 326 | 9.49 | 2.85 | | 3.32 | 0 | 0 | 0 | 11.28 |
| RF00004 | U2 | 76 | 1 | 184 | 215 | 5.95 | 1.12 | | 5.29 | 0 | 0 | 0 | 10.01 |
| RF00234 | glmS | 8 | 3 | 181 | 303 | 7.98 | 1.83 | | 4.36 | 0 | 0 | 0 | 11.22 |
| RF00168 | Lysine | 33 | 17 | 180 | 223 | 5.71 | 1.45 | | 3.94 | 0 | 0 | 0 | 15.98 |
| RF00380 | ykoK | 35 | 3 | 168 | 192 | 4.33 | 1.23 | | 3.52 | 0 | 0 | 0 | 13.10 |
| RF00003 | U1 | 46 | 6 | 159 | 184 | 4.14 | 0.89 | | 4.63 | 0 | 0 | 0 | 11.24 |
| RF00025 | Telomerase-cil | 10 | 2 | 157 | 188 | 3.89 | 1.00 | | 3.88 | 2 | 0 | 2 | 13.97 |
| RF00002 | 5_8S_rRNA | 62 | 1 | 151 | 183 | 3.44 | 0.97 | | 3.55 | 0 | 0 | 0 | 11.28 |
| RF00379 | ydaO-yuaA | 31 | 4 | 147 | 227 | 4.38 | 1.63 | | 2.69 | 0 | 0 | 0 | 12.25 |
| RF00067 | U15 | 9 | 3 | 146 | 178 | 2.71 | 0.98 | | 2.76 | 0 | 0 | 0 | 11.11 |
| RF00029 | Intron_gpII | 7 | 11 | 141 | 276 | 4.75 | 1.32 | | 3.59 | 1 | 0 | 1 | 11.08 |
| RF00015 | U4 | 25 | 1 | 141 | 187 | 3.66 | 1.04 | | 3.53 | 1 | 0 | 1 | 13.46 |
| RF00096 | U8 | 5 | 1 | 135 | 177 | 2.98 | 0.93 | | 3.20 | 0 | 0 | 0 | 11.56 |
| RF00080 | yybP-ykoY | 20 | 33 | 129 | 173 | 3.05 | 1.13 | | 2.69 | 1 | 0 | 1 | 10.78 |
| RF00114 | S15 | 10 | 1 | 117 | 138 | 1.78 | 0.60 | | 2.99 | 0 | 0 | 0 | 13.12 |
| RF00020 | U5 | 29 | 3 | 115 | 139 | 2.06 | 0.74 | | 2.80 | 0 | 0 | 0 | 13.64 |
| RF00059 | THI | 228 | 8 | 109 | 222 | 3.34 | 1.46 | | 2.29 | 0 | 0 | 0 | 13.66 |
| RF00504 | gcvT | 109 | 5 | 102 | 199 | 2.37 | 1.40 | | 1.70 | 0 | 0 | 0 | 13.40 |
| RF00167 | Purine | 33 | 4 | 99 | 119 | 1.49 | 0.57 | | 2.62 | 0 | 0 | 0 | 13.02 |
| RF00169 | SRP_bact | 46 | 15 | 96 | 120 | 1.47 | 0.65 | | 2.26 | 0 | 0 | 0 | 11.58 |
| RF00055 | snoZ37 | 5 | 1 | 94 | 117 | 1.14 | 0.53 | | 2.16 | 1 | 0 | 1 | 13.96 |
| RF00019 | Y | 15 | 1 | 94 | 128 | 1.42 | 0.73 | | 1.94 | 1 | 0 | 1 | 14.25 |
| RF00033 | MicF | 8 | 1 | 93 | 114 | 1.28 | 0.51 | | 2.50 | 0 | 0 | 0 | 13.18 |
| RF00213 | snoR38 | 7 | 3 | 88 | 147 | 1.36 | 0.70 | | 1.94 | 0 | 0 | 0 | 16.07 |
| RF00054 | U25 | 5 | 1 | 87 | 107 | 0.96 | 0.46 | | 2.09 | 1 | 0 | 1 | 16.66 |
| RF00206 | U54 | 12 | 1 | 81 | 115 | 0.94 | 0.53 | | 1.76 | 1 | 0 | 1 | 15.80 |
| RF00104 | mir-10 | 9 | 2 | 73 | 94 | 0.85 | 0.52 | | 1.64 | 2 | 0 | 2 | 16.13 |
| RF00005 | tRNA | 1080 | 19 | 73 | 127 | 1.35 | 0.48 | | 2.81 | 5 | 1 | 4 | 12.62 |
| RF00170 | msr | 5 | 3 | 70 | 112 | 0.86 | 0.45 | | 1.92 | 3 | 0 | 3 | 13.49 |
| RF00163 | Hammerhead_1 | 65 | 1 | 68 | 233 | 1.60 | 0.90 | | 1.77 | 0 | 0 | 0 | 15.82 |
| RF00031 | SECIS | 11 | 24 | 64 | 87 | 0.69 | 0.42 | | 1.63 | 13 | 2 | 11 | 14.58 |
| RF00165 | Corona_pk3 | 10 | 1 | 63 | 80 | 0.55 | 0.31 | | 1.78 | 1 | 0 | 1 | 14.72 |
| RF00066 | U7 | 28 | 2 | 62 | 85 | 0.59 | 0.34 | | 1.73 | 0 | 0 | 0 | 14.23 |
| RF00008 | Hammerhead_3 | 82 | 1 | 55 | 101 | 0.71 | 0.44 | | 1.61 | 0 | 0 | 0 | 14.71 |
| RF00037 | IRE | 36 | 1 | 28 | 45 | 0.17 | 0.12 | | 1.39 | 1 | 0 | 1 | 17.64 |
| MER statistics summed across all families | | | | | |  | | | | 96 | 5 | 91 | N/A |
| Summary MER statistics (using one threshold for all families) | | | | | |  | | | | 114 | 3 | 111 | 16.38 |
| average timing statistics | | | | | | 10.02 | 1.64 | 4.21 | |  | | | |
| total timing statistics | | | | | | 510.86 | 83.48 | 6.12 | |  | | | |

Table 5: **Rfam benchmark families with timing and MER statistics.** “W” = window length, maximum size of a hit per family, calculated as dmax(0). Running times for standard (non-banded) and QDB () searches are given for each family, in CPU-hours per Mb. The MER threshold (“thr” column) is the bit score for a given family at which the sum of false positives (“FP”) and false negatives (“FN”) is minimized. “MER” = minimum error rate, FP+FN at threshold. In the row labeled “Summary MER statistics”, these are derived from a single score threshold in a ranked list of all hits across all families. All statistics are for infernal version 0.72 in local alignment mode.
